# Spontaneous Cardiac Tamponade in a Patient with Severe Hemophilia A: A Case Report

**DOI:** 10.5811/cpcem.64682

**Published:** 2026-10-20

**Authors:** Teenu Singh, Sachin Wali, Shreyas Gutte, Afzal Azim, Banani Poddar

**Affiliations:** Sanjay Gandhi Postgraduate Institute of Medical Sciences, Department of Critical Care Medicine, Lucknow, India

**Keywords:** hemophilia A, cardiac tamponade, factor VIII deficiency, case report

## Abstract

**Introduction:**

Hemophilia A is an X-linked recessive bleeding disorder characterized by deficiency of factor VIII. The clinical features of this disease are hemorrhage and can be life-threatening when major organ systems are involved.

**Case Report:**

We report the case of a 13-year-old boy with severe hemophilia A and factor VIII inhibitors who presented with fever and septic arthritis, later developing refractory shock with tachyarrhythmia. Despite initial suspicion of sepsis-related myocarditis, point-of-care ultrasound (POCUS) revealed massive hemorrhagic pericardial effusion with tamponade physiology. Recombinant factor VIIa was transfused as hemostatic rescue therapy; the patient was subsequently started on fresh frozen plasma and emicizumab. Ultrasound-guided pericardiocentesis resulted in rapid hemodynamic recovery.

**Conclusion:**

This case highlights the importance of considering obstructive shock in unstable hemophilia patients and demonstrates that timely POCUS-based diagnosis and factor-supported pericardiocentesis can be lifesaving.

## INTRODUCTION

Hemophilia A is an X-linked recessive bleeding disorder caused by a quantitative or qualitative deficiency of factor VIII (FVIII) resulting from heterogeneous mutations in the FVIII gene located on chromosome Xq28.^1^ The disease is characterized by spontaneous or trauma-related bleeding, with clinical manifestations largely determined by the severity of the deficiency. Bleeding most commonly involves joints (hemarthroses) and muscles (hematomas), although life-threatening hemorrhage can occur when critical organs such as the central nervous system or cardiovascular system are affected.

Hemorrhagic pericardial effusion leading to cardiac tamponade is a rare but potentially fatal complication of hemophilia. Early diagnosis and regular FVIII replacement therapy may help prevent such bleeding events. However, management becomes particularly challenging in patients who develop FVIII inhibitor antibodies because conventional high-dose FVIII replacement may be ineffective. Additional challenges include the bleeding risk associated with invasive procedures such as pericardiocentesis and the lack of standardized management guidelines for hemorrhagic pericardial effusion in patients with hemophilia and other bleeding disorders.^2^,^3^

Furthermore, hemophilia-related hemorrhagic pericardial effusion is exceedingly uncommon; a PubMed search identified only seven previously reported cases over the past several decades. We report a case of severe hemophilia A and concurrent FVIII inhibitor antibodies presenting with hemorrhagic pericardial effusion causing cardiac tamponade. This case highlights the diagnostic and therapeutic challenges associated with this rare but life-threatening complication.

## CASE REPORT

A 13-year-old boy diagnosed with hemophilia A and low-level FVIII inhibitors, regularly receiving FVIII replacement, presented to the hospital with a history of fever and swelling in the right knee with associated pain for four days and was admitted to the medical genetics ward (day 1). There was no history of trauma. On examination, vitals were as follows: heart rate (HR), 96 beats per minute; blood pressure (BP), 110/70 mm Hg; respiratory rate, 20 breaths per minute; and peripheral oxygen saturation (SpO_2_), 99% on room air. His right knee was swollen with tenderness over the anterior aspect, with localized warmth and generalized restriction in range of movement. Dorsalis pedis artery and posterior tibial artery pulses of his right lower extremity were present. Laboratory investigations showed the following: hemoglobin, 7.4 g/dL (reference range, 11–14 g/dl); total leukocyte count (TLC), 17 × 10^9^/L (4–10 × 10^9^/L); platelets, 148 × 10^9^/L (150–450 × 10^9^/L); activated partial thromboplastin time (aPTT), 52 seconds (24–39 seconds); and international normalized ratio (INR), 1.25 (0.9–1.3). Ultrasonography of his right knee showed a fluid collection in the anterior aspect, which was hypoechoic with internal echoes suggestive of knee abscess.

The patient underwent incision and drainage in the operating room, and was managed along with empiric antibiotics, FVIII concentrate and monoclonal antibody emicizumab (day 5). There was reduction in pain and swelling, but intermittent fever for four hours a day up to 99.5 °F persisted; TLC count increased to 20 × 10^9^/L, and antibiotics were modified as per culture reports (day 12).

On subsequent days, patient’s fever intensified and became associated with dyspnea (day 15). On examination, his vitals were as follows: HR, 198 beats per minute; BP, 80/50 mm Hg; respiratory rate, 40 breaths per minute; and SpO2, 98% on room air. Electrocardiogram (ECG) was suggestive of narrow complex tachycardia (supraventricular tachyarrhythmia). The patient was started on oxygen supplementation, and two doses of intravenous (IV) adenosine were given over 3 seconds (total of 18 mg) with no reduction in HR. Following this, IV amiodarone (150 mg) followed by 1mg/kg/hr continuous infusion was started. He was also initiated on IV noradrenaline (norepinephrine) infusion at a rate of 0.6 μg/kg/min and noninvasive positive pressure ventilation. Possibility of myocarditis secondary to sepsis was considered, and he was transferred to intensive care unit-level of care. On examination, he had thready pulses with pulsus paradoxus, muffled heart sounds, an ECG showing electrical alternans (Image), and severe metabolic acidosis on arterial blood gas.

*CPC-EM Capsule*

What do we already know about this clinical entity?

*Hemophilia A can rarely cause spontaneous hemopericardium and cardiac tamponade, a life-threatening complication requiring urgent recognition and treatment.*

What makes this presentation of disease reportable?

*Spontaneous hemopericardium caused tamponade in a child with severe hemophilia A, FVIII inhibitors, and sepsis-associated coagulopathy.*

What is the major learning point?

*Consider cardiac tamponade in unexplained shock in hemophilia; bedside point-of-care ultrasound (POCUS) enables rapid diagnosis, while hemostatic support permits lifesaving drainage.*

How might this improve emergency medicine practice?

*POCUS should be used early in unexplained shock in hemophilia, enabling rapid recognition of tamponade and timely, factor-supported pericardiocentesis.*

Suspecting obstructive shock, we performed point-of-care ultrasound (POCUS), which demonstrated a massive pericardial effusion (3.5 cm) between the visceral and parietal pericardium at end-diastole, with right ventricular diastolic collapse in the subcostal four-chamber view (Video). IIn consultation with a hematologist and a cardiologist, the decision was made to relieve the tamponade with pericardiocentesis. Laboratory results during this time showed the following: hemoglobin, 9.5 g/dL; TLC, 20.9 × 10^9^/L; platelets, 10.9 × 10^9^/L, INR, 4.0; and aPTT, 61 seconds. Liver function test showed the following: total bilirubin, 2.84 mg/dL (reference range, 0.3–1.3 mg/dl); direct bilirubin, 1.51 mg/dL (< 0.3 mg/dl); alanine aminotransferase, 34 u/L (5–55 u/L); aspartate aminotransferase, 225 u/L (15–60 u/L); and alkaline phosphatase, 170 u/L (142–335 u/L). The patient was transfused 250 μg/kg of recombinant FVII activated (Factor FVIIa), and subsequently started on 90 μg emicizumab and 15 ml/kg of fresh frozen plasma intravenously. After 30 minutes, ultrasound-guided percutaneous drainage of the tamponade through a subxiphoid approach was done, and a flexible 8-French catheter was left in the pericardial space as a pericardial drain.

An initial 300 mL of fluid was drained from the effusion, which was hemorrhagic in nature, suggestive of hemopericardium due to hemophilia A. There was improvement in hemodynamic parameters and signs of tamponade resolved on repeat ultrasound. Pericardial fluid sent for microbiological examination was negative for infectious etiology. On the first day, a total of 900 mL hemorrhagic fluid drained, with subsequent days decreasing in volume consistently (second day 250 mL, third day 180 mL, fourth day 30 mL). The catheter was removed on the seventh day following the pericardiocentesis. During this period the patient received seven units of packed red blood cells, seven units fresh frozen plasma, and six pooled cryoprecipitate units. The patient continued to be managed with FVIII (1,000 U twice a day) and emicizumab (90 μg daily). Repeat echocardiography showed no pericardial effusion, and the patient was transferred to ward on day 28.

## DISCUSSION

This case illustrates a unique clinical scenario involving severe hemophilia A with low-titer FVIII inhibitors complicated by sepsis-associated coagulopathy and hemorrhagic pericardial effusion causing cardiac tamponade. The diagnosis was particularly challenging because refractory shock and tachyarrhythmia initially raised suspicion of myocarditis, while the underlying tamponade physiology was revealed by POCUS. The coexistence of FVIII inhibitors and sepsis-related coagulopathy substantially increased the risk of bleeding and complicated hemostatic management. Despite these challenges, timely diagnosis, hemostatic optimization and ultrasound-guided pericardiocentesis resulted in rapid hemodynamic recovery. This case highlights the importance of maintaining a high index of suspicion for cardiac tamponade in critically ill patients with hemophilia and demonstrates that factor-supported pericardiocentesis can be performed safely when appropriate hemostatic measures are instituted.

Hemorrhagic pericardial effusion involves collection of bloody fluid within the pericardial cavity and can evolve into cardiac tamponade. Common etiologies include thoracic trauma, complications of ischemic heart disease, infection, autoimmune conditions, and anticoagulants. Of the reported hemophilia cases with hemorrhagic pericardial effusion, there was a preceding illness such as rheumatic fever, viral fever, pr chest trauma that could have precipitated hemorrhagic pericardia effusion.^3^–^5^ In our case, hemophilia A with FVIII inhibitors was the primary predisposing factor, whereas the coagulopathy secondary to sepsis and mild liver dysfunction could have exacerbated the bleeding. Hence, this underscores the importance of early source control in sepsis and prompt treatment of coagulopathy in patients with hemophilia A.

Cardiac tamponade results from accumulation of pericardial fluid causing impaired cardiac filling and reduced cardiac output.^6^ In our patient, dyspnea, tachyarrhythmia, and refractory shock were initially attributed to sepsis and possible myocarditis, delaying consideration of an obstructive cause of shock. The diagnosis of tamponade is often challenging because classic findings such as Beck triad are frequently absent.^2^,^7^ Also in critically ill patients, overlapping shock states may further obscure the diagnosis. Therefore, unexplained hemodynamic deterioration, escalating vasopressor requirements, or shock disproportionate to the apparent severity of sepsis should prompt evaluation for cardiac tamponade. In such situations, POCUS is invaluable because it can rapidly identify pericardial effusion and tamponade physiology at the bedside, allowing timely diagnosis and intervention. In this case, POCUS was instrumental in distinguishing obstructive shock from presumed septic shock and directly guided definitive management.

Primary management of cardiac tamponade is by drainage of the pericardial effusion; even a small amount of fluid drainage can help relieve intrapericardial pressure and tamponade effects. Although several relative contraindications exist, a retrospective review of 1,196 patients who underwent drainage by a cardiologist in the presence of thrombocytopenia (platelet count < 50 × 10^9^/L) and coagulopathy (INR > 1.5), did not show significant association with increased major bleeding.^7^ Goz et al reported a case of an 11-year-old boy in which 1,000 U of factor VIII was given and pericardial window was created through a mini thoracotomy in the left fourth intercoastal space with drainage of 200 cc of fluid. There was no bleeding seen in cardiac structure or main vessels, and he was discharged on the fifth postoperative day.^8^

In our case, prior to pericardiocentesis, FVIIa and fresh frozen plasma were given for treatment of coagulopathy. The International Society on Thrombosis and Haemostasis clinical practical guidelines suggest that individuals with severe hemophilia A with FVIII inhibitors who are undergoing invasive procedures should be treated with bypassing agents, either FVIIa or activated prothrombin complex concentrate.^9^ In the present case, given that emicizumab prophylaxis was ongoing, FVIIa was preferred because a combination of activated prothrombin complex concentrate and emicizumab could lead to thrombotic complications.^9^ Importantly, in hemodynamically unstable patients with tamponade physiology, hemostatic optimization and procedural planning should occur simultaneously, and lifesaving pericardial drainage should not be delayed once hemostatic measures have been initiated.

Hemorrhage in patients with hemophilia can be effectively prevented with regular factor replacement therapy that maintains adequate physiologic FVIII levels. In the case reported by Zayani et al, the FVIII dose was increased to 50 IU/kg every eight hours during an episode of hemorrhagic pericardial effusion. In contrast, in the 11-year-old male reported by Goz et al, bleeding was successfully managed with a lower-intensity regimen of 50 IU/kg every 12 hours.^6^,^8^ The goal of prophylaxis in hemophilia A is to prevent spontaneous bleeding. Prophylactic dose usually ranges from 10–40 IU/kg three times weekly, which is achieved by maintaining trough FVIII levels around 1–3%, with higher target intensities ≥ 3–5% individualized for patients with higher bleeding severity. In patients with FVIII inhibitors, emicizumab provides an effective alternative with reduced treatment burden given its weekly schedule.^9^,^10^ However, there are no standardized guidelines regarding optimal factor replacement during hemorrhagic pericardial effusion, and management should be individualized based on clinical severity and response to therapy. 9

A practical approach to hemorrhagic cardiac tamponade in patients with hemophilia and FVIII inhibitors involves four key steps: 1) prompt recognition of tamponade should be considered in patients with hemodynamic instability by confirmation through POCUS; 2) hemostatic rescue should be initiated immediately using appropriate bypassing agents such as recombinant FVIIa or activated prothrombin complex concentrate and correction of associated coagulopathy with blood products should be undertaken as clinically indicated; 3) urgent pericardiocentesis should be performed once initial hemostatic support has been established; and 4) continued postprocedural hemostatic support and serial echocardiographic surveillance are essential to prevent recurrent bleeding and re-accumulation of pericardial fluid.

A limitation of this report is the inability to definitively determine the exact precipitating mechanism of the hemorrhagic pericardial effusion. Although severe hemophilia A with FVIII inhibitors was likely the primary contributor, concurrent sepsis-associated coagulopathy and mild hepatic dysfunction may have exacerbated the bleeding tendency and contributed to the development of hemorrhagic cardiac tamponade.

## CONCLUSION

Acute hemorrhagic pericardial effusion and resulting cardiac tamponade occurring spontaneously in patients with hemophilia is a rare condition and no consensus exists for optimal management. This case gives insight regarding factor replacement in the presence of FVIII inhibitors and sepsis-induced coagulopathy with a consideration for need of urgent pericardiocentesis.

## Supplementary Information

Video. Point-of-care ultrasound (subcostal four-chamber view) demonstrating a large circumferential pericardial effusion. Yellow arrow indicates the pericardial effusion, while a red arrow highlights diastolic collapse of the right ventricle, consistent with cardiac tamponade physiology.

## Figures and Tables

**Image:**
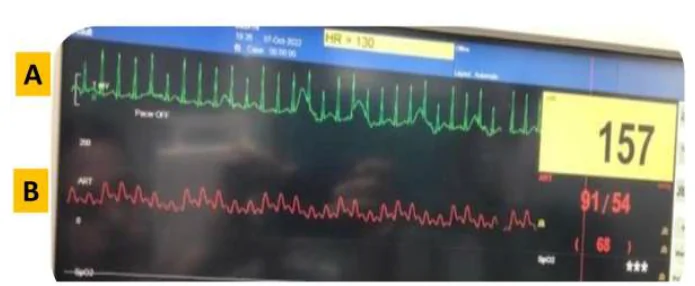
Patient monitor **A**) electrocardiogram strip showing electrical alternans, and **B**) arterial blood pressure waveform showing pulsus paradoxus.
